# Corneal Biomechanics Differences Between Chinese and Caucasian Healthy Subjects

**DOI:** 10.3389/fmed.2022.834663

**Published:** 2022-02-25

**Authors:** Riccardo Vinciguerra, Robert Herber, Yan Wang, Fengju Zhang, Xingtao Zhou, Ji Bai, Keming Yu, Shihao Chen, Xuejun Fang, Frederik Raiskup, Paolo Vinciguerra

**Affiliations:** ^1^Humanitas San Pio X Hospital, Milan, Italy; ^2^Department of Ophthalmology, University Hospital Carl Gustav Carus, Dresden, Germany; ^3^Tianjin Eye Hospital, Tianjin Key Laboratory of Ophthalmology and Visual Science, Nankai University Affiliated Eye Hospital, Tianjin, China; ^4^Clinical College of Ophthalmology, Tianjin Medical University, Tianjin, China; ^5^Beijing Tongren Eye Center, Beijing Tongren Hospital, Beijing Ophthalmology and Visual Sciences Key Lab, Capital Medical University, Beijing, China; ^6^EYE & ENT Hospital of Fudan University, Shanghai, China; ^7^BAI JI Ophthalmology, Chongqing, China; ^8^Zhongshan Ophthalmic Center, Sun Yat-Sen University, Guangzhou, China; ^9^Eye Hospital, Wenzhou Medical University, Zhejiang, China; ^10^Shenyang Aier Eye Hospital, Shenyang, China; ^11^Department of Biomedical Sciences, Humanitas University, Milan, Italy; ^12^IRCCS Humanitas Research Hospital, Rozzano, Italy

**Keywords:** biomechanics, cornea, keratoconus, CBI, IOP (intraocular pressure)

## Abstract

**Purpose:**

The aim of this study was to evaluate the difference between Caucasian and Chinese healthy subjects with regards to Corvis ST dynamic corneal response parameters (DCRs).

**Methods:**

Two thousand eight hundred and eighty-nine healthy Caucasian and Chinese subjects were included in this multicenter retrospective study. Subsequently, Chinese eyes were matched to Caucasians by age, intraocular pressure (IOP), and Corneal Thickness (CCT) using a case-control matching algorithm. The DCRs assessed were Deformation Amplitude (DA) Applanation 1 velocity (A1v), integrated radius (1/R), deformation amplitude ratio (DAratio), stiffness parameter at applanation 1 (SPA1), ARTh (Ambrósio's Relational Thickness to the horizontal profile), and the novel Stress Strain Index (SSI).

**Results:**

After age-, CCT-, and IOP- matching, 503 Chinese were assigned to 452 Caucasians participants. Statistical analysis showed a statistical significant difference between Chinese and Caucasian Healthy subjects in the values of SPA1 (*p* = 0.008), Arth (*p* = 0.008), and SSI (*p* < 0.001). Conversely, DA, A1v, DAratio, and 1/R were not significantly different between the two ethnical groups (*p* > 0.05).

**Conclusion:**

We found significant differences in the values of the DCRs provided by the Corvis ST between Chinese and Caucasian healthy subjects.

## Introduction

Ethnical differences in ocular metrics are well-known since many years and include central corneal thickness (1), corneal curvature (2), anterior chamber depth (3), and axial length (4).

In the last years, corneal biomechanics showed to play an important role for the diagnosis and management of keratoconus (5–9) post refractive surgery ectasia (10), cross-linking effect (11), measurement of intraocular pressure (12, 13), and glaucoma (14, 15).

Two instruments are commercially available to measure corneal biomechanics, the Ocular Response Analyzer (ORA, Reichert Inc., Depew, NY) (16) which measures corneal deformation during a bi-directional applanation method induced by an air jet, and produces appraisals of corneal hysteresis and corneal resistance factor, together with a set of 36 waveform-derived parameters (17–19). The Corvis ST (OCULUS Optikgeräte GmbH; Wetzlar, Germany) evaluates the reaction of the cornea to an air puff *via* an ultra-high speed (UHS) Scheimpflug camera, and uses the acquired image sequence to generate estimates of IOP and deformation response parameters (DCRs) (20).

The native software of the Corvis ST includes normative values for each DCRs which were derived from a mixed south American and Caucasian population (21). Very few population studies have been published with regards to DCRs values in other ethnical populations (22–24) and none of them evaluated the difference between two different ethnical groups.

The aim of this study was to assess the difference between Caucasian and Chinese healthy subjects with regards to Corvis ST DCRs.

## Methods

Two thousand eight hundred and eighty-nine healthy Caucasian and Chinese patients were included in this multicenter retrospective study. Caucasian subjects were recruited from Vincieye Clinic in Milan, Italy and from the Department of Ophthalmology, University Hospital Carl Gustav Carus, Technical University, Dresden, Germany. Conversely, Chinese participants were included from Beijing Tongren Eye Center, Beijing Tongren Hospital, Capital Medical University, Beijing; Shenyang Aier Eye Hospital, Shenyang, Zhongshan Ophthalmic Center, Sun Yat-Sen University, Guangzhou; EYE&ENT Hospital of Fudan University, Shanghai; Eye Hospital, Wenzhou Medical University, Zhejiang; BAI JI Ophthalmology, Chongqing, and Tianjin Eye Hospital,Tianjin.

Each Institutional review board (IRB) either ruled that approval was not required for this record review study or specifically approved the study. The research was conducted according to the ethical standards set in the 1964 Declaration of Helsinki, revised in 2000. All patients signed an informed consent before using their data in the study. All subjects underwent to a complete ophthalmic examination, including the Corvis ST and Pentacam exams. The inclusion criteria of this study were the existence in the database of a Corvis ST and Pentacam exam, a Belin Ambrosio Enhanced Ectasia Index total deviation (BAD-D) <1.6 and a signed informed consent. Exclusion criteria were any earlier ocular surgery or disease, any concurrent or previous glaucoma or hypotonic therapies. All exams with the Corvis ST were acquired by the same experienced technicians and captured by automatic release to ensure the absence of user dependency. Only Corvis ST exams with quality score “OK” were included in the analysis. Only 1 eye per subject was randomly included in the database to exclude the bias of the relationship between bilateral eyes that could influence the analysis result.

The parameters that were included in the analysis were the following: Deformation Amplitude Deformation Amplitude (DA, the largest displacement of corneal apex in the anterior-posterior direction at the moment of highest concavity) Applanation 1 velocity (A1v the velocity of corneal apex at first applanation), integrated radius (1/R the amount of the corneal concave state over the time between applanation 1 and applanation 2), deformation amplitude ratio (DAratio, the ratio between the central deformation and the average of peripheral deformation determined at 2.00 mm), stiffness parameter at applanation 1 [SPA1 is defined as the resultant pressure at inward applanation divided by the corneal displacement (25)], ARTh (Ambrósio's Relational Thickness to the horizontal profile), which is based on the thickness profile in the temporal-nasal direction (26) and the novel Stress Strain Index [SSI, which measures biomechanical behavior of the cornea without influence of corneal thickness and intraocular pressure (27)]. Additonally, the bIOP intraocular pressure estimate was included as a corrected value that is less influenced by age, corneal thickness and other DCR parameters (28).

### Statistical Analysis

The statistical analysis was performed with SPSS version 27 (IBM Corp. in Armonk, NY, USA). In this study Chinese eyes were matched by age, bIOP, and Central Corneal Thickness (CCT) using a case-control matching algorithm provided by SPSS (29).

Descriptive statistics were calculated for the DCRs described previously, additionally, differences between data were evaluated with analysis of variance (ANOVA). The chosen level of significance was *p* < 0.05.

## Results

After age-, CCT- and bIOP- matching, 503 Chinese were assigned to 452 Caucasians participants. Mean age-, CCT- and bIOP of Chinese were 30.2 ± 6.8 years, 542.7 ± 29.7 μm, and 15.8 ± 2.1 mmHg, respectively, whereas, Caucasians showed 31.1 ± 6.8 years, 547.9 ± 31.8 μm, and 15.6 ± 2.1 mmHg of mean values.

Table 1 shows mean baseline characteristics of the two groups.

**Table 1:** Baseline and demographic data of the study population.

**Parameter**	**Caucasians**	**Chinese**
Age	31.1 ± 0.3	30.2 ± 0.3
CCT	547.9 ± 31.8	542.7 ± 29.7
bIOP	15.5 ± 2.2	15.7 ± 2.1
Eye (%Right)	45.1%	49.9%

**Figure 1:**
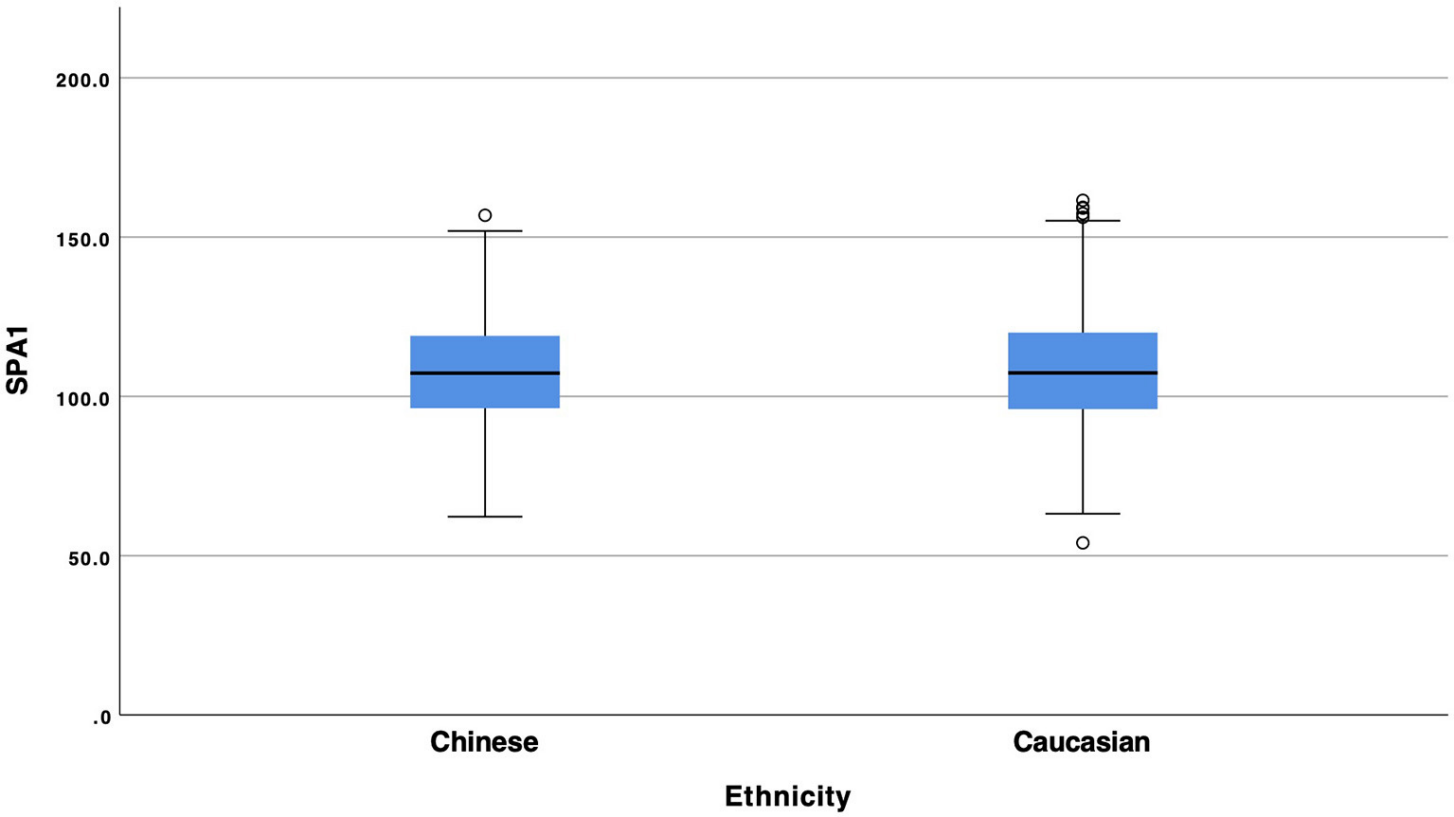
Box and whiskers plot of SP-A1 of Chinese and Caucasian population.

**Figure 2:**
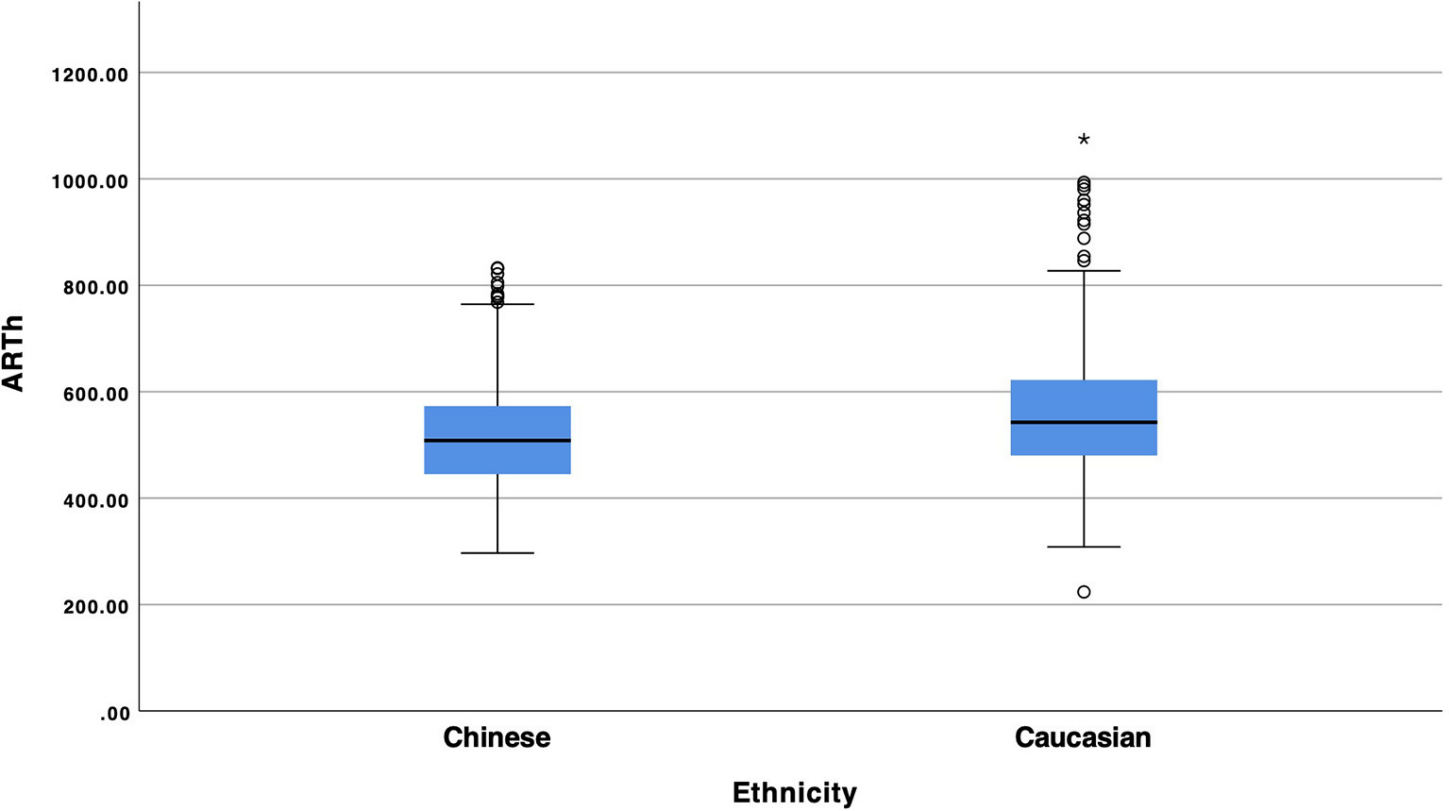
Box and whiskers plot of Arth of Chinese and Caucasian population.

**Figure 3:**
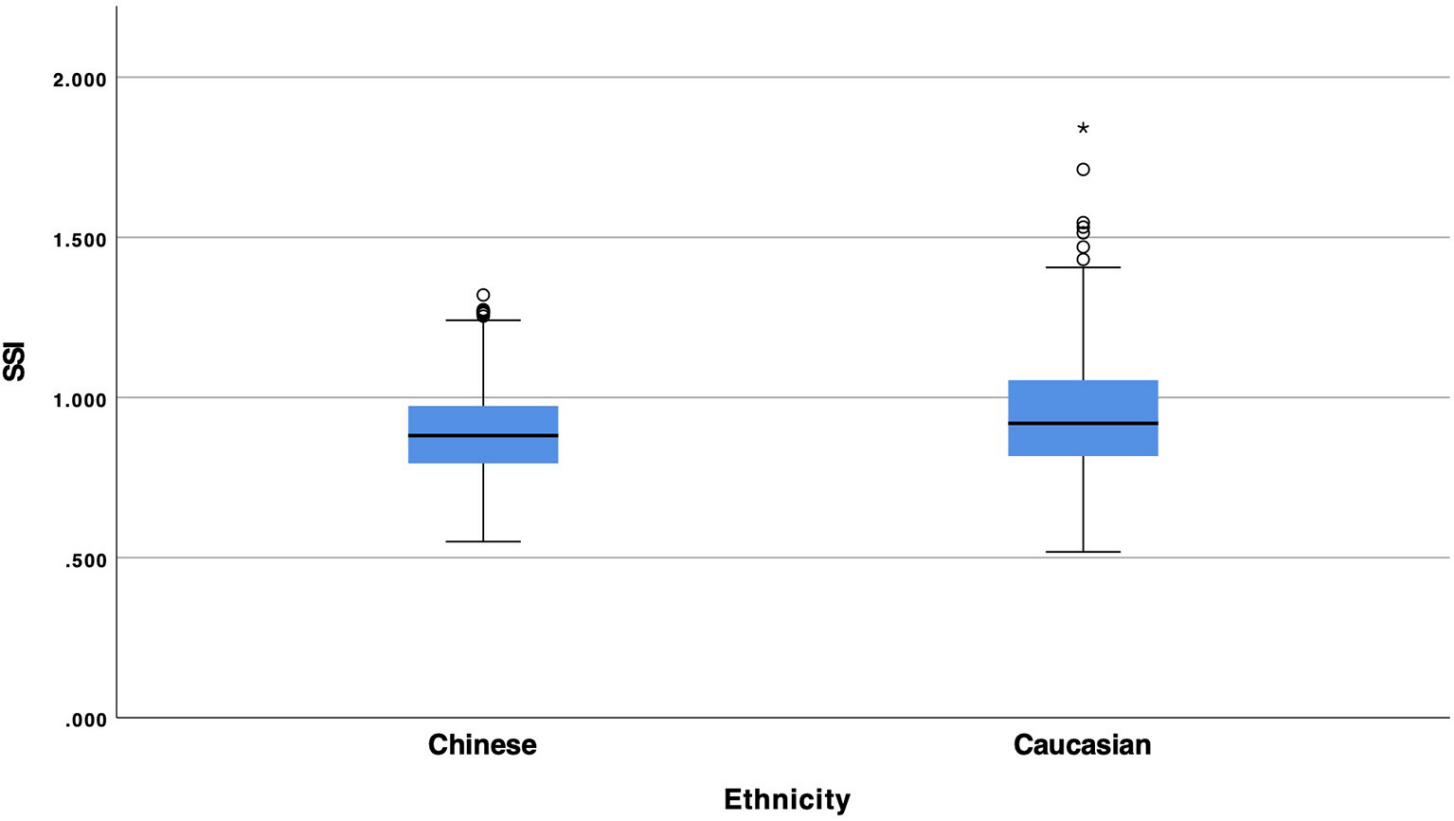
Box and whiskers plot of SSI of Chinese and Caucasian population.

Statistical analysis showed a statistical significant difference between Chinese and Caucasian Healthy subjects in the values of SPA1 (Figure 1, *p* = 0.008), Arth (Figure 2, *p* = 0.008) and SSI (Figure 3, *p* < 0.001). Conversely, DA (*p* = 0.674), A1v (*p* = 0.373), DAratio (*p* = 0.656), and 1/R (*p* = 0.184) were not significantly different between the two ethnical groups. Table 2 provides more details of the results of the ANOVA.

**Table 2:** Number of cases, mean, standard deviation and p values of Corvis DCRs between Chinese and Caucasian population.

**Parameters**		***N***	**Mean**	**Standard deviation**	***p*-value**
DA	Chinese	503	1.08485	0.102870	0.674
	Caucasian	452	1.07484	0.100243	
SPA1	Chinese	503	107.844	16.0351	**0.008**
	Caucasian	452	108.456	18.9433	
DARatio	Chinese	503	4.5317	0.45785	0.656
	Caucasian	452	4.3025	0.46629	
ARTh	Chinese	503	518.7264	100.13527	**0.008**
	Caucasian	452	563.2689	120.32852	
1/R	Chinese	503	8.5521	1.05437	0.184
	Caucasian	452	8.2790	1.17436	
A1v	Chinese	503	0.1517	0.01951	0.793
	Caucasian	452	0.1468	0.01883	
SSI	Chinese	503	0.88984	0.137459	**<0.0001**
	Caucasian	452	0.94163	0.186990	

*Bold means significant values*.

## Discussion

The evaluation of Ethnical variances in ocular metrics is not only important for the pure scientific knowledge but, more importantly, because a difference between two ethnicities could play a role in disease diagnosis.

The main finding of this study was the evidence that there is a significant difference in the values of the DCRs of the Corvis ST between Chinese and Caucasian population, more in details SPA1 and SSI which are pure biomechanical parameters and Arth which measures the thickness profile in the temporal-nasal direction.

It should be noted that these results are not due to the possible variance in age, IOP or corneal thickness between the two groups as they were specifically matched for these confounding factors. We decided not to match the patients for sex and refractive error to avoid decreasing too much the number of patients and we concentrated on age, IOP and CCT which are the most significant confounding factor for corneal biomechanics measurement (26).

It is the first time, to the authors' knowledge, that a large multicenter study was able to show a significant difference in corneal biomechanics (either Corvis ST or ORA) between two ethnical populations.

The importance of these results could be extremely high particularly in the sensitivity and the specificity of the Corvis Biomechanical Index (CBI) which includes all the three indices which were found to be different and was created basing on Caucasian and South American populations (8). We expect that this difference could play a significant role when screening a Chinese patient for refractive surgery that could lead potentially to false positives.

It is worth mentioning though only few studies on Chinese keratoconus patients assessed the sensitivity and specificity values of the CBI when compared to the original publication and they showed similar results (30, 31).

Further work of this group will focus on assessing the sensitivity and the specificity of CBI in Chinese keratoconus and to evaluate whether there is a need to improve the algorithm for this specific ethnic group.

In conclusion, we found significant differences in the values of the DCRs provided by the Corvis ST between Chinese and Caucasian healthy subjects. The presence of a case-control matching confirms this finding and excludes the influence of age, IOP, and CCT as confounding factors.

## Data Availability Statement

The datasets presented in this article are not readily available because it was not possible to be made public due to local laws.

## Ethics Statement

Each Institutional Review Board (IRB) either ruled that approval was not required for this record review study or specifically approved the study. Written informed consent from the patients/participants was not required to participate in this study in accordance with the national legislation and the institutional requirements.

## Author Contributions

All authors listed have made a substantial, direct, and intellectual contribution to the work and approved it for publication.

## Conflict of Interest

RV and PV are consultants for OCULUS Optikgeräte GmbH. OCULUS Optikgeräte GmbH did not take part in the design, analysis, or interpretation of the results. The remaining authors declare that the research was conducted in the absence of any commercial or financial relationships that could be construed as a potential conflict of interest.

## Publisher's Note

All claims expressed in this article are solely those of the authors and do not necessarily represent those of their affiliated organizations, or those of the publisher, the editors and the reviewers. Any product that may be evaluated in this article, or claim that may be made by its manufacturer, is not guaranteed or endorsed by the publisher.
